# Tetrahydrocurcumin extends life span and inhibits the oxidative stress response by regulating the FOXO forkhead transcription factor

**DOI:** 10.18632/aging.100396

**Published:** 2011-12-08

**Authors:** Lan Xiang, Yukiko Nakamura, Young-Mi Lim, Yasutoyo Yamasaki, Yumi Kurokawa-Nose, Wakako Maruyama, Toshihiko Osawa, Akira Matsuura, Noboru Motoyama, Leo Tsuda

**Affiliations:** ^1^ Department of Cognitive Brain Sciences, National Center for Geriatrics and Gerontology, Obu, Aichi, 474-8511, Japan; ^2^ Animal Models of Aging, National Center for Geriatrics and Gerontology, Obu, Aichi, 474-8511, Japan; ^3^ Department of Applied Molecular Bioscience, Graduate School of Bioagricultural Sciences, Nagoya University, Nagoya 464-8601, Japan; ^4^ Department of Nanobiology, Graduate School of Advanced Integration Science, Chiba University, Chiba 263-8522, Japan; ^5^ Current address: College of Biosystems Engineering and Food Science, Zhejiang University, Hangzhou 310029, China

**Keywords:** tetrahydrocurcumin, Drosophila, NIH-3T3-FOXO4 cell line, anti-aging, FOXO, resveratrol

## Abstract

The O-type forkhead domain transcription factor (FOXO) is involved in many biological processes such as aging, the oxidative stress response, and growth regulation. FOXO activity is tightly controlled within cells. In particular, growth factor signaling pathways and the oxidative stress response can both stimulate nuclear translocation of this transcription factor. Here, we show that tetrahydrocurcumin (THC), a curcumin metabolite, regulates the oxidative stress response and aging via FOXO. In NIH3T3 cells, THC induced nuclear accumulation of FOXO4, a member of the FOXO family of transcription factors, by inhibiting phosphorylation of protein kinase B (PKB)/Akt. In Drosophila melanogaster, THC attenuated the oxidative stress response, an effect that was blocked in a foxo mutant background. THC also extended the life span of Drosophila under normal conditions, and loss of either foxo or Sir2 activity eliminated this effect. Based on these results, THC may regulate the aging process via an evolutionarily conserved signaling pathway that includes both foxo and Sir2.

## INTRODUCTION

The insulin/IGF-1 signaling pathway regulates aging processes in *Drosophila melanogaster, Caenorhabditis elegans*, and mammals [1, 2]. Phosphoinositide-3-kinase (PI3K) and Akt kinase are critical downstream components of the insulin/IGF-1 pathway, and regulate the transcriptional activity of O-type forkhead domain transcription factors (FOXOs) [1]. There is a large family of mammalian FOXO transcription factors, which includes FOXO1, FOXO3, FOXO4, and FOXO6 [3, 4]. These proteins are involved in many cellular events, such as cell cycle arrest, apoptosis, DNA repair, glucose metabolism, anti-oxidative stress response, and longevity [5-7].

The activity and nuclear translocation of FOXO proteins are tightly regulated by post-translational modifications, including phosphorylation, acetylation, ubiquitination, and protein/protein interactions [4]. Oxidative stress stimulates the nuclear localization of FOXO3a *via* the sirtuins (SirTs), members of the silent information regulator 2 (Sir2) family of class III histone/protein deacetylases [8, 9]. Given that Sir2 family members regulate aging, the FOXO/SirT regulatory network may be a key factor for understanding the relationship between oxidative stress and life span [9].

Curcumin (C) is a yellow dye found in the crude drug turmeric (*Curcumae Rhizoma*), which comes from the rhizome of *Curcuma longa L*. (*Zingiberaceae*). C exhibits anti-oxidative [10], anti-inflammatory [11], liver-protective [12], anti-spastic [13], anti-tumor [14], and anti-allergic [15] effects. Tetrahydrocurcumin (THC) is an active metabolite of C [10, 11]. Orally ingested C is metabolized into THC by a reductase found in the intestinal epithelium [16, 17]. THC possesses extremely strong anti-oxidant activity compared to other curcuminoids [18, 19]. The anti-oxidant role of THC has been implicated in recovery from renal injury in mice [20] and in anti-inflammatory responses [18]. However, the literature concerning the anti-aging mechanism of THC is limited to a single survival study in mice [21]. In our current study, we found that THC regulated the nuclear localization of FOXO in cultured cells and inhibited phosphorylation of protein kinase B (PKB)/Akt kinase. Furthermore, genetic analyses in *Drosophila* revealed that *foxo* and *Sir2* mediated the effects of THC on life span and the oxidative stress response. These results suggest that THC regulates the oxidative stress response and aging *via* an evolutionally conserved signaling pathway.

## RESULTS

### THC regulates nuclear localization of FOXO4 protein in NIH3T3 cells

**Figure 1:**
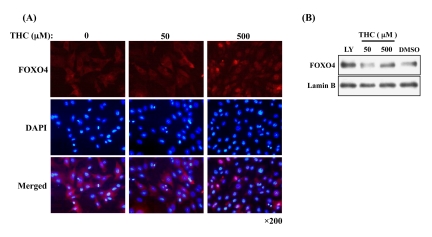
THC-induced nuclear accumulation of FOXO4 (**A**) Dose-dependent effects of THC on nuclear accumulation of FOXO4 in NIH-3T3-FOXO4 cells. Cy3 (red) and DAPI (blue) label FOXO4 and nuclei, respectively. (**B**) FOXO4 levels increase in nuclear fractions from NIH-3T3-FOXO4 cells. Lamin B was used as a nuclear marker and the Akt inhibitor LY294002 (LY) was used as a positive control. Anti-AFX (N-19) primary antibody was used in both analyses.

**Figure 2:**
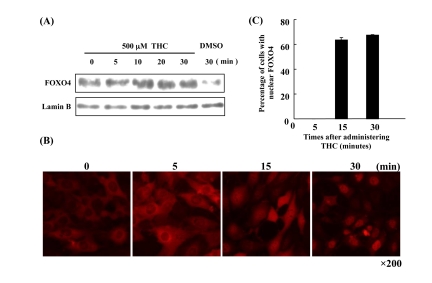
Time course of FOXO4 localization in response to THC (**A**) FOXO4 protein levels increase in the nuclear fraction of NIH-3T3-FOXO4 cells within minutes of THC treatment. Lamin B was used as a nuclear marker, DMSO as a negative control. (**B**) Time course of FOXO4 nuclear translocation in NIH-3T3-FOXO4 cells following administration of THC. Red indicates FOXO4 protein. (**C**) The number of cells with nuclear FOXO was quantified at the indicated times following THC administration.

It has been shown that THC regulates the oxidative stress response in cells [22]. Nuclear accumulation of FOXO4 is a molecular marker for activation of the oxidative stress response [23]. We asked, therefore, whether THC affects the nuclear localization of FOXO4 in NIH3T3 cells. Immunocytochemistry revealed that THC treatment increased the nuclear levels of FOXO4 in a dose-dependent manner (Figure 1A). We confirmed this result through cell fractionation experiments. Levels of FOXO4 protein in the nuclear fraction were clearly elevated by THC treatment (Figure 1B). Next, we performed a time course analysis and detected FOXO4 in the nucleus within 30 min of THC treatment (Figure 2). These results indicate that THC regulates the nuclear translocation of FOXO4.

### Effect of THC on signaling pathways that regulate FOXO4 localization in NIH-3T3 cells

To investigate the molecular mechanisms by which THC affects the nuclear localization of FOXO4, we analyzed the mitogen-activated protein kinase (MAPK) signaling pathway and the insulin-Akt signaling pathway. Both have been shown to regulate the nuclear translocation of FOXO4. Activation of ERK1/2 (a member of the MAPK subfamily) has been shown to increase the nuclear accumulation of FOXO4 [24]. We asked, therefore, whether THC affects phospho-ERK1/2 proteins in the NIH-3T3-FOXO4 cell line, and found that THC did not inhibit ERK1/2 phosphorylation (Supplemental Figure 1).

**Figure 3:**
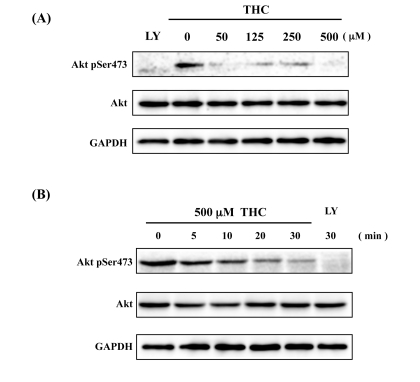
THC inhibits Akt phosphorylation Dose-dependence (**A**) and time-course (B) analyses indicate that THC inhibits phosphorylation of Akt^Ser473^. LY294002 (LY) was used as a positive control, Akt and GAPDH antibodies as loading controls.

PKB/Akt kinase, acting as a downstream component of the insulin signaling pathway, phosphorylates FOXO4, and inhibits its nuclear translocation. This activity is thought to inhibit aging processes in multi-cellular organisms [25]. We focused, therefore, on the phosphorylation state of Akt using a phospho-specific Akt antibody (Akt^Ser473^). We found that THC treatment significantly inhibited phosphorylation of Akt in a dose-dependent manner. The effects of THC on phospho-Akt were similar to those observed with the positive control, LY294002 (Figure 3A). Time-course analysis indicated that THC treatment inhibited Akt phosphorylation within 30 min (Figure 3B). This result was consistent with the time-course analysis of FOXO4 nuclear localization (Figure 2). These analyses suggest that THC promotes translocation of FOXO4 to the nucleus at least in part through inhibition of Akt phosphorylation.

### THC represses the oxidative stress response in Drosophila via foxo

**Figure 4:**
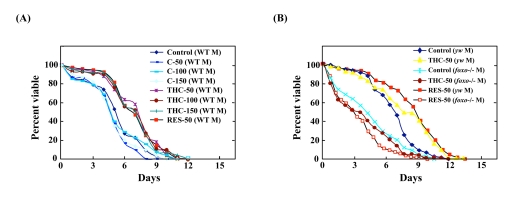
THC regulates oxidative stress response in *Drosophila* via *foxo* activity Effects of THC, RES, and C on the life span of wild-type (WT, Oregon-R) (**A**) and *foxo* mutant (*yw* background) (**B**) flies under oxidative stress conditions. Oxidative stress was induced by adding 7.5 mM paraquat to the food. Life span studies were carried out at 25°C with newly eclosed males (M) in each group (20 per vial; 3-4 vials were counted). Survivors were transferred every 2-3 days to fresh vials containing THC, RES, or C (50-150 μM). The statistical analysis of the data is summarized in Table 1.

To determine whether FOXO mediates the effect of THC *in vivo*, we turned to *Drosophila* as a model organism. It has been shown that *Drosophila foxo* is involved in the oxidative stress response [26]. We first investigated, therefore, whether THC affects the oxidative stress response in *Drosophila*. Flies were fed 7.5 mM paraquat, a superoxide-generating agent. Under these conditions, all control flies were dead after 12 d of treatment. THC treatment led to a significant restoration of survival (~28%) (Figure 4A, Table 1). Interestingly, C did not increase tolerance to oxidative stress (Figure 4A, Table 1). Given that both C and THC have anti-oxidant activity, the effect of THC on life span may not be attributable to an effect on scavenging of reactive oxygen species [18, 19].

As shown above, THC regulated FOXO nuclear translocation in mammalian cells. We investigated, therefore, the effects of THC on the oxidative stress response in a *foxo*-null mutant background. Although THC extended the life span of wild-type *Drosophila*, it could not extend the life span of *foxo*-null flies under oxidative stress conditions (Figure 4B, Table 1). These results suggest that THC regulates the oxidative stress response *via foxo*.

**Table 1:** Results of *Drosophila* life span experiment

				Mean lifespan		Log-rank test		
Trial	Food	Strain	Treatment	N	days	χ^2^	P	% change
1	7.5 mM (Paraquat)	*Oregon-R* (Male)	Control	99	7			
			RES-50	95	8	10.47	0.0012	+14.3*
			THC-50	97	9	12.24	0.0001	+28.5*
			THC-100	106	9	9.556	0.0020	+28.5*
			THC-150	91	8	7.448	0.0063	+14.3*
			C-50	106	6	2.728	0.0986	− 14.3^ns^
			C-100	99	6	1.179	0.2775	− 14.3^ns^
			C-150	103	6	3.141	0.0764	− 14.3^ns^
2	7.5 mM (Paraquat)	*yellow white foxo−/−* (Male)	Control	71	5			
			RES-50	62	5	0.570	0.4499	0.0^ns^
			THC-50	57	6	0.660	0.4147	+20.0^ns^
3	STD	*yellow white* (Female)	Control	79	47			
			THC-20	80	54	10.510	0.0012	+14.8*
			THC-50	78	57	11.509	0.0034	+21.2*
			RES-50	72	49	11.95	0.0026	+5.2*
		*yellow white* (Male)	Control	75	38			
			THC-50	80	45	11.05	0.0009	+18.4*
		*Organ-R* (Male)	Control	70	46			
			C-20	73	44	0.2584	0.6114	− 4.3^ns^
			C-150	74	44	0.2845	0.5938	− 4.3^ns^
4	STD	*foxo−/− (yw)* (Male)	Control	76	33			
			RES-50	76	25	1.559	0.2899	− 24.2^ns^
			THC-50	77	30	1.123	0.2118	− 9.1^ns^
		*d4EBP−/− (yw)* (Male)	Control	79	31			
			RES-50	70	28	7.466	0.0063	− 9.6*
			THC-50	77	33	0.029	0.8136	+6.5^ns^
5	STD	*Sir2−/df (yw)* (Male)	Control	78	32			
			RES-50	80	27	0.2766	0.0787	− 15.6^ns^
			THC-50	78	27	3.0910	0.5990	− 15.6^ns^

* indicates significant difference from control (P < 0.01)

ns represented no significant difference (P > 0.05)

STD: Standard, RES: resveratrol, THC: tetrahydrocurcumin, C: curcumin

Resveratrol (RES) is a chemical compound that activates SIRT1 deacetylase activity and suppresses the oxidative stress response in mammalian cells [27]. It has been shown that SIRT1 regulates the state of acetylation of FOXO and induces nuclear localization of FOXO [9]. Indeed, we confirmed addition of RES to the culture medium resulted in nuclear accumulation of FOXO4 (Supplemental Figure 2). We also showed that RES treatment prolonged the life span of *Drosophila* under oxidative stress conditions (Figure 4A; Table 1). RES activity under oxidative stress conditions was inhibited in the *foxo* mutant background (Figure 4B; Table 1). These results suggest that RES and THC regulate the oxidative stress response in *Drosophila* via *foxo*.

### THC extends the life span of Drosophila

**Figure 5:**
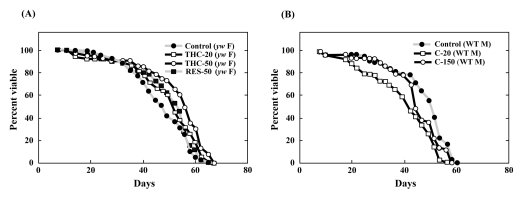
THC, but not C, extends life span in *Drosophila* under normal conditions The effect of THC and RES (**A**) was analyzed in a yw background, and C (**B**) was analyzed in Oregon-R (WT) flies. Life span studies were carried out at 25°C with newly eclosed male (M) or female (F) flies for each longevity experiment (20 per vial; 4 vials were counted). Survivors were transferred to fresh vials containing THC, RES, or C (20—150 μM) every 2—3 days. The statistical analysis of the data is summarized in Table 1.

*foxo* regulates life span through the insulin signaling pathway [1, 28, 29]. We asked, therefore, whether THC also regulates life span under normal conditions. Before performing this analysis, we determined that THC does not affect the growth or eating habits of *Drosophila* (Supplemental Figures 3, 4). Life-span analysis showed that THC extended the mean but not maximum life span in both female flies (by ~21%) (Figure 5A; Table 1). We also found that RES prolonged the life span of *Drosophila* (Figure 4A; Table 1), although the effect was smaller than that seen with THC. This result differs from the report [30], that did not detect a significant effect of RES on *Drosophila* life span.

### Foxo and d4E-BP activity is required for life-span extension by THC

**Figure 6:**
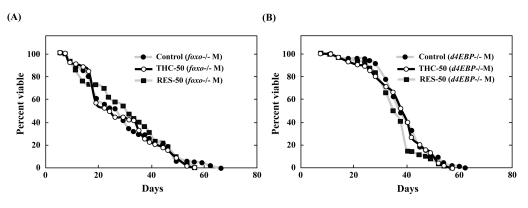
THC and RES do not extend the life span of *foxo* or *d4E-BP* mutants Effects of THC or RES (50 μM) on the life span of *foxo*−/− (**A**) or *d4E-BP*−/− (**B**) mutant *Drosophila* under natural conditions. Life span studies were carried out at 25°C with a total of around 80 newly eclosed male (M) flies (*yw* background). The statistical analysis of the data is summarized in Table 1.

We next tested the ability of THC to extend the life span of *foxo*-null mutant flies. Compared to wild-type *Drosophila*, *foxo* mutants were short-lived. THC exposure, however, did not extend the life span of these *foxo* mutants (Figure 6A; Table 1). To confirm these results, we analyzed the effect of THC on a null mutant for the gene encoding eukaryotic initiation factor 4E-binding protein (*d4E-BP*), which acts downstreamof *foxo* to mediate aging and oxidative stress responses [31-33]. THC did not alter the life span of *d4E-BP*-null mutant flies (Figure 6B; Table 1). The effect of RES on life span was similarly abrogated in both *foxo* and *d4E-BP* mutant backgrounds (Figure 6; Table 1). These data indicate that THC and RES regulate the life span of *Drosophila* through *foxo* and *d4E-BP* activity.

### Sir2 activity is required for life-span extension by THC

**Figure 7:**
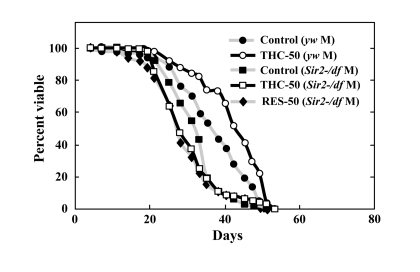
THC and RES do not extend the life span of *Sir2* mutants Effects of THC or RES (50 μM) on the life span of *Sir2-/df* mutant *Drosophila* under natural conditions. Life span studies were carried out at 25°C with a total of around 80 newly eclosed male (M) flies (*yw* background). The statistical analysis of the data is summarized in Table 1.

In addition to phosphorylation, acetylation is also known to control the nuclear localization of FOXO [9]. Sirtuin1, an NAD^+^-dependent deacetylase, plays a particularly important role in regulating the acetylation state of FOXO, and has been shown to affect the nuclear localization FOXO under oxidative stress in mammalian cells [9]. We therefore tested whether *Drosophila Sir2* was required for THC to affect life span. We found that *Sir2*-null mutants were short-lived and that THC did not extend their life span (Figure 7; Table 1). These results suggest that THC extends the *Drosophila* life span *via* a mechanism that is dependent on *foxo* and *Sir2*. Similarly, RES did not affect the life span of *Sir2* mutants, which supports the hypothesis that RES upregulates *Sir2* activity (Figure 7; Table 1).

## DISCUSSION

We present here the first evidence that the small chemical compound THC is associated with the anti-oxidative stress response and extension of life span *via* the FOXO transcription factor. Using a mammalian cell culture system, we found that THC regulated FOXO4 nuclear translocation (Figure 1). Akt may be involved in this effect, since THC treatment caused Akt dephosphorylation. Phospho-Akt normally prevents the nuclear localization of FOXO (Figure 3). To support this analysis, we found that THC extended the life span of *Drosophila* under oxidative stress conditions and that this effect was *foxo*-dependent (Figure 4). Furthermore, THC extended the life span of *Drosophila* under normal conditions, and this extension required *foxo* and *d4E-BP* activity (Figure 6). These findings support the idea that oxidative stress may correlate with life-span extension. Notably, the effect of THC on life span also seemed to depend on *Sir2* activity (Figure 7), suggesting that THC regulates aging processes *via* an evolutionally conserved regulatory network of genes that includes both *foxo* and *Sir2*.

Recently, chemical biology approaches have enabled researchers to analyze complicated biological processes such as aging using vast arrays of chemical compounds. As a result, plant-derived phenolic compounds have garnered a great deal of attention, because RES, a phenolic compound found in red wine, has been reported to extend life span in yeast, nematodes, and mice [34-36]. An important component of RES function is its ability to activate the SirTs [37-39]. Although there are indications that RES may not extend life span in *Drosophila*, our studies support the hypothesis that RES indeed regulates life span through *Sir2* activity [30] (Figure 7). We speculate that the difference between these studies may have resulted from experimental variation or the source of THC.

Here, we found that another plant-derived phenolic compound, THC, regulates animal aging and the oxidative stress response *via* specific biological networks. Both C and THC have previously been reported to display anti-oxidant effects [18, 19]. In our current study, however, only THC affected the oxidative stress response and life span in *Drosophila* (Figure 5A). These results support another previous study in which C metabolites had more potent biological effects than C itself [40]. C has also been shown to inhibit the histone-modifying enzyme CBP/p300 [41]. This may explain why C actually appeared to have a rather toxic effect in *Drosophila* (Figure 5B).

THC treatment resulted in the nuclear localization of FOXO4 and dephosphorylation of Akt (Figs. 1, 3). Akt inhibits nuclear localization of FOXO4 and plays a key role in regulating its activity. These results suggest that THC regulates nuclear localization of FOXO protein by affecting either acetylation or ubiquitination. Our genetic analyses using *Drosophila* suggest that the relationship between THC and FOXO is evolutionarily conserved. Notably, THC activity seemed to depend on *d4E-BP*, a downstream target of *foxo* (Figure 6). This result suggests that THC affects the *foxo-d4E-BP* pathway, consistent with a recent study showing that the *foxo-d4E-BP* system regulates animal life span by affecting proteostasis [33]. We also observed that THC activity depended on *Sir2* (Figure 7). Although we cannot exclude the possibility that the *foxo*, *d4E-BP*, and *Sir2* mutations cause non-specific cellular toxicity that negatively affects life span (and that THC regulates this effect), our combined results in cultured mammalian cells and *Drosophila* suggest that THC may be involved in specific events that regulate an organism's life span.

Caloric restriction is known to have an anti-oxidative effect and to extend life [42]. The life-extending effect of caloric restriction is associated with increased level and activity of *Drosophila* Sir2 histone deacetylase and its mammalian ortholog, SIRT1 [35, 43]. SIRT1 also seems to be involved in the oxidative stress response by regulating the nuclear localization of FOXO [27]. We demonstrated here that both RES and THC depend on *foxo* and *Sir2* to extend the life span of *Drosophila*. Although we do not know whether THC and RES share a common target in order to regulate longevity, we expect that THC and RES regulate very similar down-stream effectors. THC may regulate FOXO, which might be involved in Sir2-dependent life-span extension.

Finally, it should be noted that THC-treated mice also survive for an extended period of time [21]. Interestingly, it was shown that the average mouse life span, but not the maximum life span, is extended by THC treatment. As THC also increased the average but not maximum life spans of Drosophila (Figure 4), these studies suggest that the effect of THC on longevity may be an evolutionarily conserved process.

## MATERIALS AND METHODS

### Curcumin, tetrahydrocurcumin, and resveratrol

C and THC were kindly provided by House Foods Industry (Okinawa, Japan). Resveratrol (RES) was purchased from Wako Pure Chemical Industries (Osaka, Japan).

### FOXO4 immunostaining in NIH-3T3 cells

NIH-3T3 cells (3 × 10^4^) were seeded in 48-well plates and incubated for 2 days. Cells were incubated 10 min in 500 μl of fresh culture medium and then 2.5 μl of 20 μM insulin growth factor (IGF) was added to each well. Subsequently, 0, 50, 200, or 500 μM resveratrol (RES) or THC was added. The Akt inhibitor LY294002 was used as a positive control (5 μl of 2 mM LY294002 per well). Cells were incubated for 30 min at room temperature and then 250 μl of 3% paraformaldehyde in PBS was added for 15 min at room temperature to attach the cells to the wells. The supernatant was gently removed with a pipette, and 200 μl of 0.5% Triton/CSK-buffer (Cytoskeletal buffer: 20 mM HEPES, 50 mM NaCl, 3 mM MgCl_2_ and 300 mM Sucrose) was added to each well. Cells were washed with PBS-T (PBS+0.05% Tween 20), then 100 μl of anti-AFX (N-19) antibody (diluted 1:100 in PBS-T) was added to each well, and cells were incubated for 1 h at room temperature. Cells were washed twice in PBS-T, then 100 μl of CY3-labeled secondary antibody (diluted 1:200 in PBS-T) was added to each well, and cells were incubated at room temperature in the dark for 1 h. Cells were washed twice with PBS-T, 200 μl of PBS containing DAPI (Roche Applied Science, USA) was added to each well (1μg/mL), and cells were observed using a fluorescence microscope (Axio version II, Carl Zeiss Inc. Germany).

### Western blot analysis

NIH-3T3 cells (1 × 10^6^ cells) were seeded in 90-cm dishes and incubated for 2 d. RES and THC were then added at doses of 0, 50, 200, and 500 μM for the appropriate times. For a positive control, 5 μl of 2 mM LY294002 was added. To prepare total protein samples, cells were harvested in RIPA buffer containing 1% phosphatase and 2% protease inhibitor. After the centrifugation the supernatant was collected and used as the total protein sample. To prepare nuclear and cytoplasmic protein samples, cells were harvested in PBS buffer and washed twice. After the centrifugation the pellet was resuspended in 400 μl of NP-40 buffer (20 mM Tris-HCl, 137 mM NaCl, 10% glycerol, 1% nonidet P-40, 2 mM EDTA). After incubation on ice for 5 min, samples were centrifuged 5 min at 2500 rpm (600× *g*) at 4°C. The supernatant was removed and used as the cytoplasmic protein sample. Pellets were washed twice with NP-40 buffer and resuspended in 200 μl high-salt buffer at 4°C for 30 min. After centrifugation at 15,000 rpm (20400× *g*) at 4°C for 5 min, the supernatant was collected and used as the nuclear protein sample. The protein concentration assay was performed using a BAC kit (Thermo Science, Pierce Company, USA). SDS-PAGE was performed using 30 μg protein, and proteins were transferred onto a PVDF membrane. The membrane was incubated with the appropriate primary antibodies followed by horseradish peroxidase-conjugated secondary antibodies, and the antigen was visualized using a chemiluminescent substrate (Amersham, GE Healthcare, Tokyo, Japan). Primary antibodies used for immunoblotting were anti-AFX1 (goat polyclonal; Santa Cruz Biotechnology, California, USA), anti-phospho-FOXO4 (Ser193) (rabbit polyclonal; Santa Cruz Biotechnology, California, USA), anti-Akt (rabbit polyclonal) and anti-phospho-Akt (Ser473) (Cell Signaling Technology, Boston, USA), mouse anti-human lamin B (Oncogene Research Products, CA, USA), rabbit anti-GAPDH (Abcam, Tokyo, Japan), and anti-β-tubulin (Sigma, St. Louis, USA). Secondary antibodies were horseradish peroxidase-conjugated anti-goat IgG, anti-mouse IgG, and anti-rabbit IgG (Amersham, GE Healthcare, Tokyo, Japan).

### Drosophila strains, medium, and life span assay

The wild-type *D. melanogaster* strain was Oregon-R, and *yellow white* flies (*y^1^,w^1118^*) were used as a second control line. To create *foxo*-null mutant flies, *yw*; *FRT82B, foxo^25^/TM6B* was crossed with *yw*; *foxo^21^/TM6B*. Both maleand female progeny were analyzed in these experiments. To create *Sir2*-null mutant flies, *yw*; *Sir2^17^/CyO* was crossed with *yw*; *Df(2L)BSC30/CyO*. *Foxo*, *Sir2* and *d4E-BP^null^* lines were kindly provided by Drs. Hafen, Helfand and Lasko, respectively.

*Drosophila* medium consisted of 0.7% agar, 10% glucose, 4.5% corn powder, and 4% dry yeast. C and THC in EtOH were added to melted aliquots of medium at final concentrations of 0, 20, 50, and 150 μM (5% EtOH). EtOH alone was used as a control. Fresh medium was prepared weekly. For survival assays, newly eclosed flies were maintained at 20 flies/vial on standard laboratory food at 25°C. Flies were transferred to fresh vials every 2-3 days and scored for survival. Each experiment was conducted with at least 200 flies of each genotype. All the analyses of life span are summarized in Table 1.

### Anti-oxidative stress analysis

To induce the oxidative stress response in *Drosophila*, adult flies (1 week old) were exposed to 7.5 mM paraquat. Twenty flies were starved in vials containing 2 ml of 1% agar for 6 h before paraquat treatment. Flies were then transferred to vials containing 1% agar with 7.5 mM paraquat (methyl viologen, Sigma, St. Louis, USA) and 5% sucrose. Data are presented as mean ± standard error.

### Statistical analysis

Significant differences between groups in all experiments were determined by Kaplan-Meier survival analysis using the biostatistics software GraphPad Prism 5. Curves were compared using the log-rank test. A p value < 0.05 was considered significant.

## SUPPLEMENTAL FIGURES

Supplemental Figure 1 THC does not affect MAPK activation Dose-dependent effects of THC on MAPK phospho-rylation in NIH-3T3-FOXO4 cells. Anti-p44/42 or phospho-p44/42 was used. Anti-GAPDH was used as a loading control. LY: LY294002, an Akt inhibitor.

Supplemental Figure 2 RES induces nuclear accumulation of FOXO4 in NIH3T3 cell (**A**) Dose-dependent effects of RES on FOXO4 nuclear localization. (**B**) RES increases the level of FOXO4 in the nuclear fraction from NIH-3T3-FOXO4 cells. LY290042 (LY) and DMSO were used as positive and negative controls, respectively. Anti-GAPDH was used as a loading control. (**C**) Dose-dependent effects of RES on Akt phosphorylation (Ser473) are shown. LY was used as a positive control. Anti-Akt and Anti-GAPDH were used as loading controls.

Supplemental Figure 3 THC does not affect larval growth Compared to 5% EtOH controls, THC treatment (50 μM) did not affect puparium formation in *Drosophila* (see Supplemental Materials and Methods).

Supplemental Figure 4 THC treatment does not affect the eating habits of *Drosophila* EtOH (5%) or THC (50 μM in 5% EtOH) was mixed with [^32^P]dCTP and fed to 14-d wild-type (Oregon-R) virgin males or females (20 animals/vial, totally 60 flies). The uptake of [^32^P]dCTP was measured by scintillation counting for 4 min/sample (see Supplemental Materials and Methods).

## Abbreviations

THC: tetrahydrocurcumin
C: curcumin
ROS: reactive oxygen species
FOXO: O-type forkhead domain transcription factor
MnSOD: Mn superoxide dismutase
RES: resveratrol
DAPI: 4′,6-diamidino-2-phenylindole dihydrochloride
CR: caloric restriction
PKB: protein kinase B)
IGF: insulin growth factor
