# Supplementary figures and images for: Tetrahydrocurcumin extends life span and inhibits the oxidative stress response by regulating the FOXO forkhead transcription factor
Source: Aging (Albany NY). 2011 Dec 8;3(11):1098–109. doi: 10.18632/aging.100396 (PMC3249455; doi:10.18632/aging.100396)

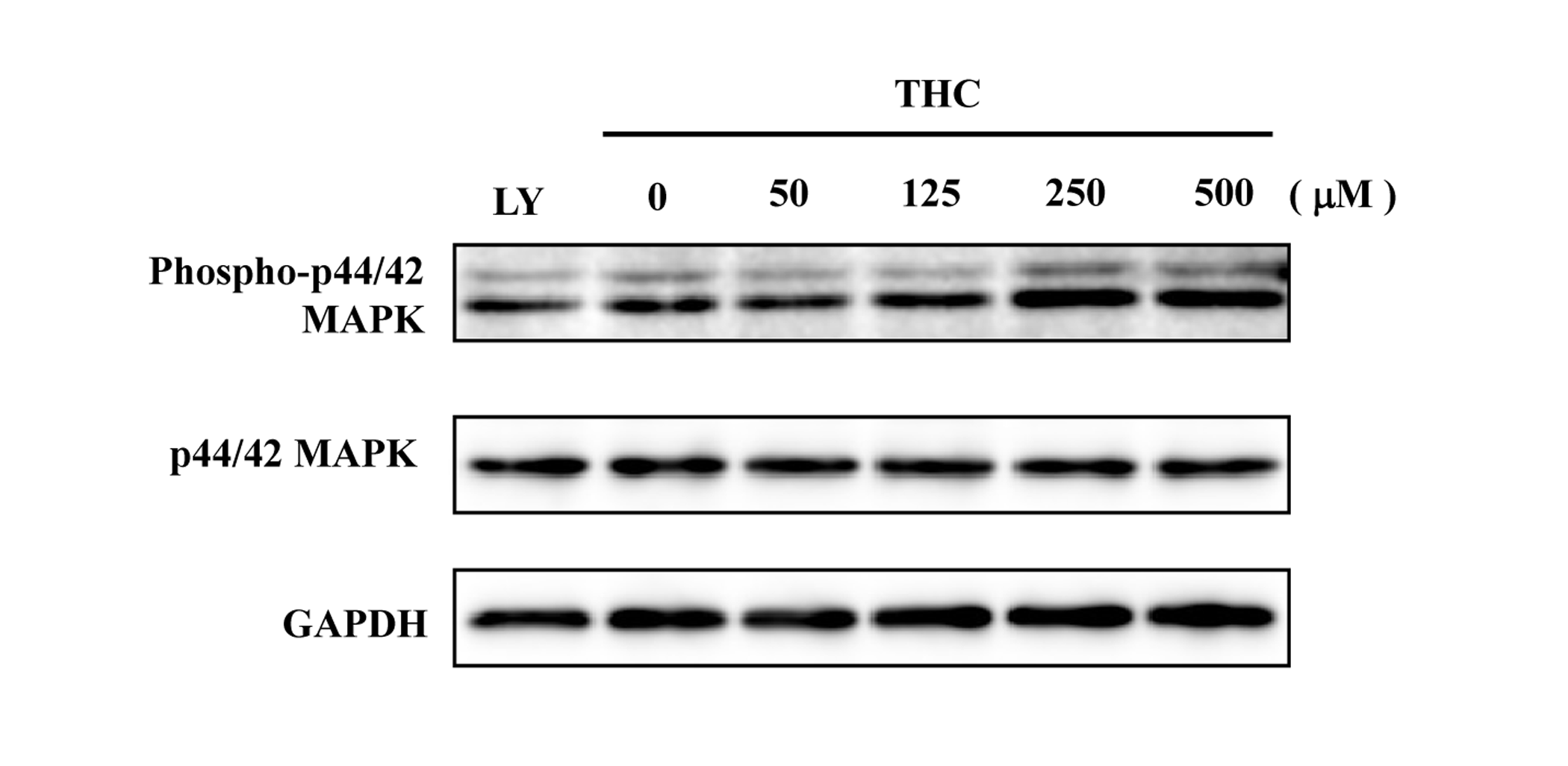

Supplement: Supplemental Figure 1 — Dose-dependent effects of THC on MAPK phospho-rylation in NIH-3T3-FOXO4 cells. Anti-p44/42 or phospho-p44/42 was used. Anti-GAPDH was used as a loading control. LY: LY294002, an Akt inhibitor. [file aging-03-1098-s001.tif]

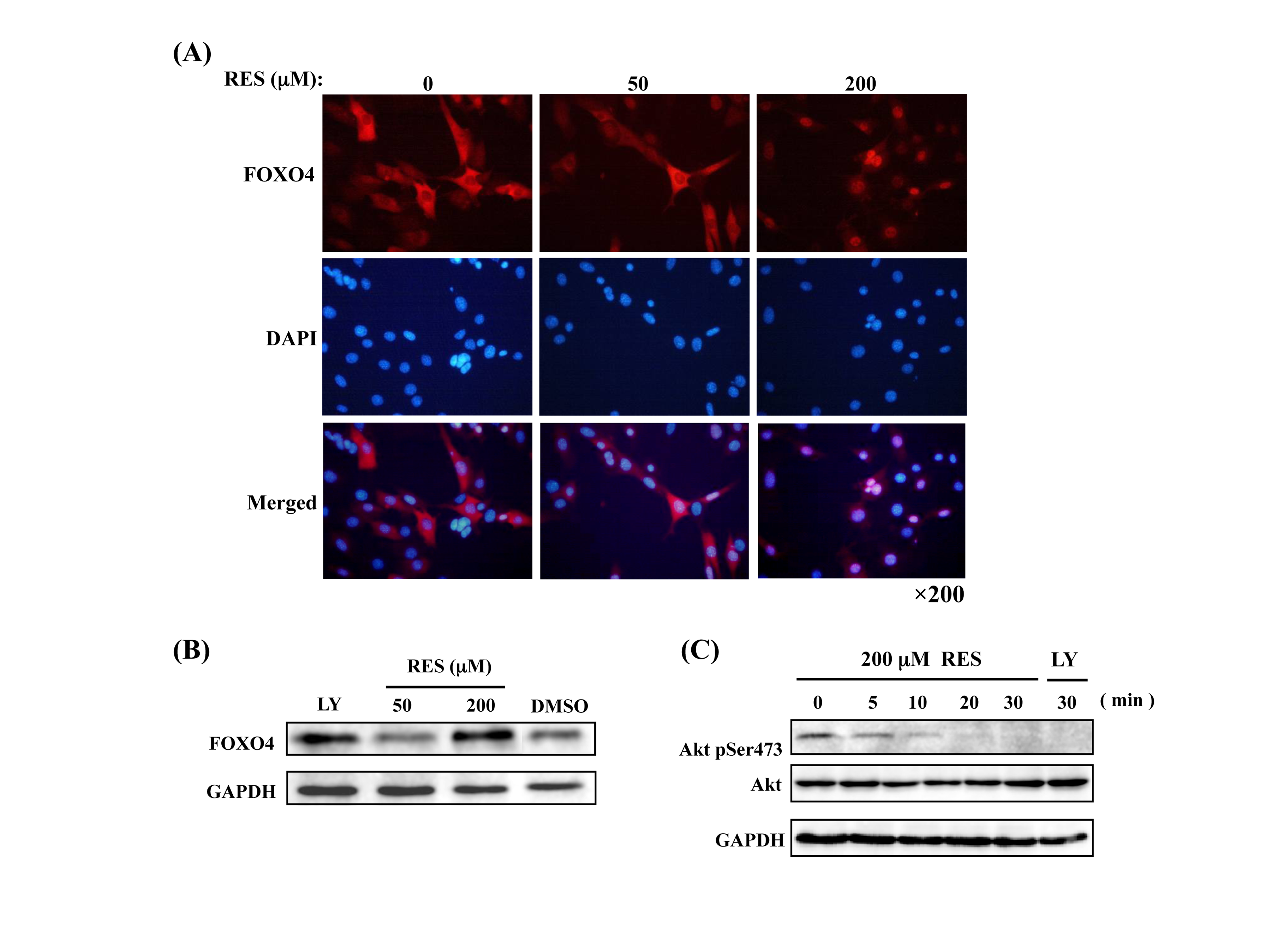

Supplement: Supplemental Figure 2 — (A) Dose-dependent effects of RES on FOXO4 nuclear localization. (B) RES increases the level of FOXO4 in the nuclear fraction from NIH-3T3-FOXO4 cells. LY290042 (LY) and DMSO were used as positive and negative controls, respectively. Anti-GAPDH was used as a loading control. (C) Dose-dependent effects of RES on Akt phosphorylation (Ser473) are shown. LY was used as a positive control. Anti-Akt and Anti-GAPDH were used as loading controls. [file aging-03-1098-s002.tif]

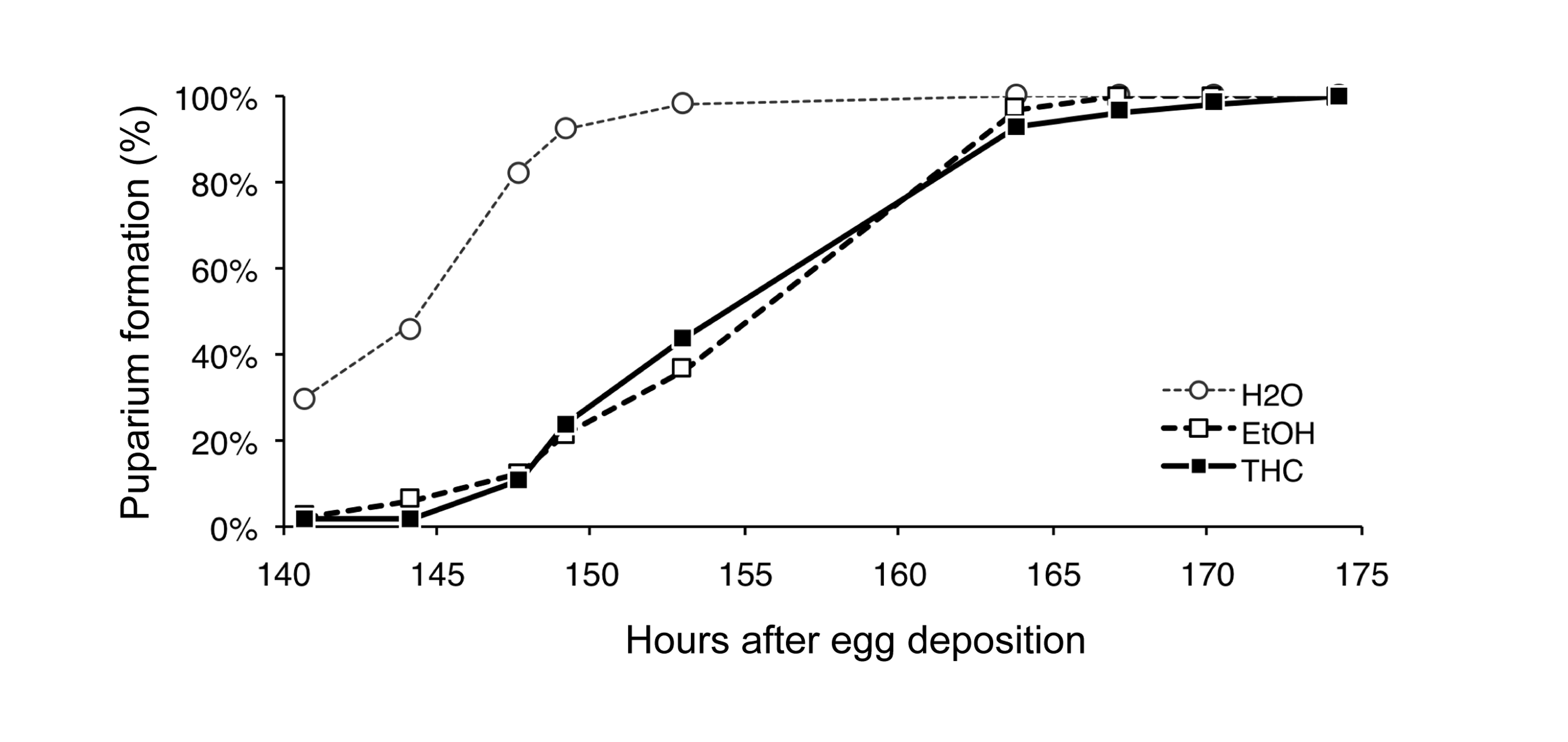

Supplement: Supplemental Figure 3 — Compared to 5% EtOH controls, THC treatment (50 μM) did not affect puparium formation in Drosophila (see Supplemental Materials and Methods). [file aging-03-1098-s003.tif]

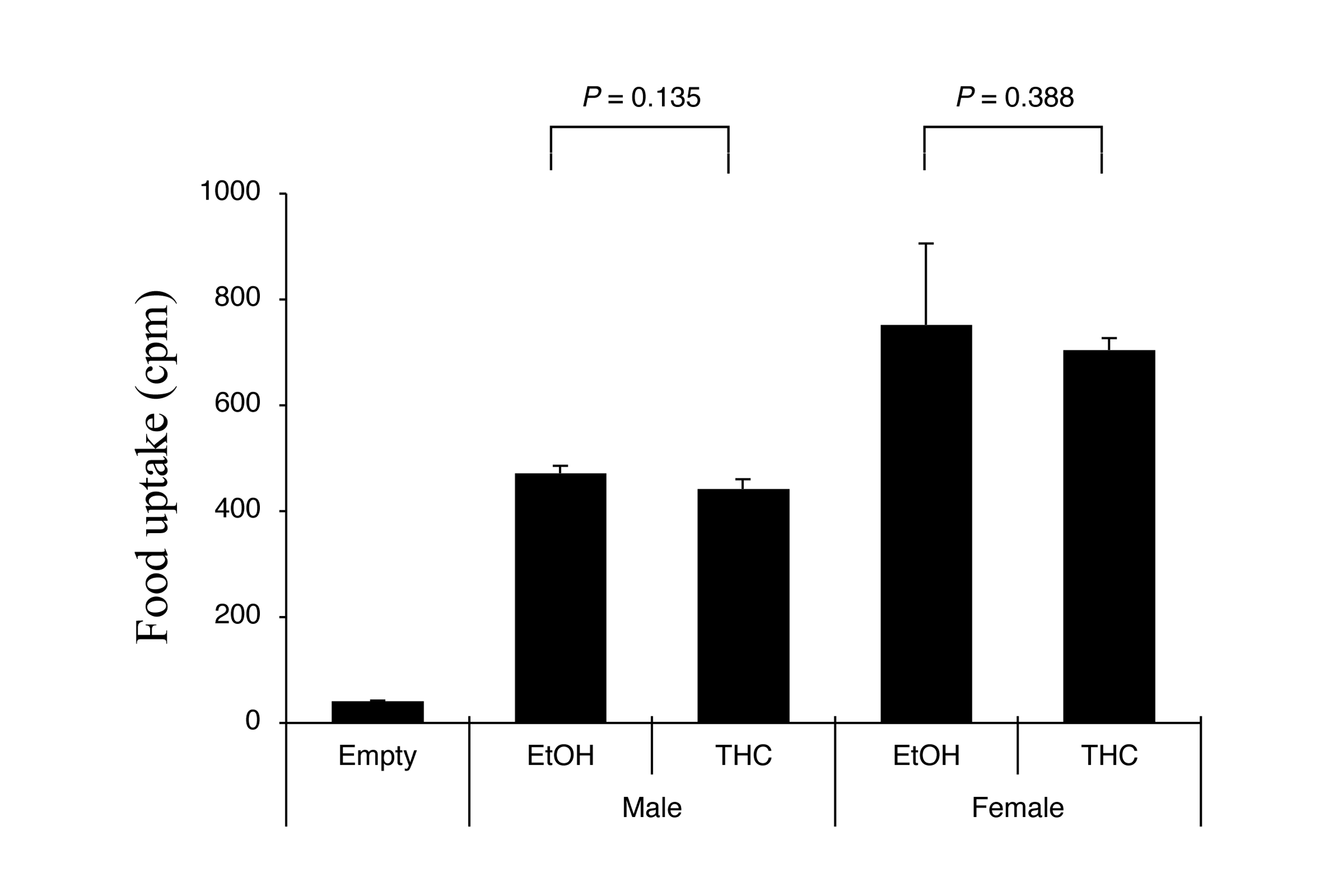

Supplement: Supplemental Figure 4 — EtOH (5%) or THC (50 μM in 5% EtOH) was mixed with [32P]dCTP and fed to 14-d wild-type (Oregon-R) virgin males or females (20 animals/vial, totally 60 flies). The uptake of [32P]dCTP was measured by scintillation counting for 4 min/sample (see Supplemental Materials and Methods). [file aging-03-1098-s004.tif]
